# Das Erlernen digitaler Gesundheitskompetenz im schulischen Kontext: Ergebnisse einer repräsentativen Befragung von Schülerinnen und Schülern in Deutschland

**DOI:** 10.1007/s00103-024-03991-6

**Published:** 2024-12-04

**Authors:** Denise Renninger, Lisa Stauch, Lisa Fischer, Anja Hartmann, Pia Rangnow, Kevin Dadaczynski, Orkan Okan

**Affiliations:** 1 Department of Health and Sport Sciences, TUM School of Medicine and Health, TUM Health Literacy Unit WHO Collaborating Centre for Health Literacy, Technische Universität München (TUM), Uptown München-Campus D, Georg-Brauchle-Ring 60/62, 80992 München, Deutschland; 2 WHO Collaborating Centre for Health Literacy, TUM School of Medicine and Health, Technische Universität München (TUM), München, Deutschland; 3 Fachbereich Gesundheitswissenschaften, Hochschule Fulda, Fulda, Deutschland; 4 Institut für Gesundheitswissenschaften, Universität zu Lübeck, Lübeck, Deutschland; 5 Department für Gesundheitswissenschaften, Hochschule für Gesundheit Bochum, Bochum, Deutschland; 6 Zentrum für angewandte Gesundheitswissenschaften, Leuphana Universität Lüneburg, Lüneburg, Deutschland

**Keywords:** Digitale Gesundheitskompetenz, Medienkompetenz, Schule, Gesundheitsinformationen, Sekundarstufe, Lernen, Digital health literacy, Media literacy, School, Health information, Secondary school, Learning

## Abstract

**Hintergrund:**

Digitale Informationsquellen bieten Jugendlichen schnellen Zugang zu gesundheitsbezogenen Informationen. Schulen sind ideal, um die digitale Gesundheitskompetenz (dGK) zu fördern und Schülerinnen und Schülern (SuS) einen sicheren Umgang mit solchen Informationen zu ermöglichen. Ziel dieses Beitrags ist es, erste Ergebnisse einer repräsentativen Studie zum Erlernen von dGK in Schulen mit Blick auf soziodemografische und sozioökonomische Unterschiede vorzustellen.

**Methoden:**

Durchgeführt wurde eine Querschnittsstudie mit 1448 SuS (9 bis 18 Jahre) in Deutschland im Rahmen des Projekts DURCHBLICKT!. Über bivariate und multivariate Analysen wurden Unterschiede und Zusammenhänge im Erlernen der dGK und Geschlecht, Alter, Migrationshintergrund sowie subjektiven Sozialstatus überprüft.

**Ergebnisse:**

Etwa 50 % der SuS gaben an, dGK (eher) nicht in der Schule erlernt zu haben. Der Chi-Quadrat-Test zeigt signifikante Unterschiede in Bezug auf Geschlecht, Alter, Migrationshintergrund und Sozialstatus. Regressionsanalysen zeigen, dass besonders das Alter und der subjektive Sozialstatus bedeutende Faktoren für das Erlernen der dGK sind.

**Diskussion:**

Die hohe Zahl der SuS, die dGK nicht in der Schule erlernen, ist besorgniserregend, vor allem im Hinblick auf ihre oft geringe dGK. Die Ergebnisse verdeutlichen die Notwendigkeit gezielter Bildungsstrategien – insbesondere solche, die auf Geschlecht und sozioökonomischen Status abgestimmt sind –, um dGK zu fördern und soziale Ungleichheiten zu verringern.

## Abstract (en)

**Background:**

Digital information sources provide adolescents with quick access to health-related information. Schools are ideal for promoting digital health literacy and enabling students to handle such information safely. The aim of this paper is to present initial results from a representative study on learning digital health literacy in schools, with a focus on sociodemographic and socioeconomic differences.

**Methods:**

The cross-sectional study was conducted in Germany with 1448 students (aged 9 to 18 years) as part of the DURCHBLICKT! project. Bivariate and multivariate analyses were used to examine differences and relationships in the learning of digital health literacy in school and gender, age, migration background, and subjective social status.

**Results:**

Approximately 50% of students reported that they had not (or only to a limited extent) learned digital health literacy in school. The chi-square test reveals significant differences in terms of gender, age, migration background, and social status. Regression analyses indicate that age and subjective social status are significant factors for acquiring digital health literacy.

**Discussion:**

The high number of students who do not learn digital health literacy in school is concerning, especially given their often low levels of digital health literacy. The results highlight the need for targeted educational strategies—especially those tailored to gender and socioeconomic status—to promote digital health literacy and reduce social inequalities.

## Hintergrund

Das Internet ist aus dem Alltag von jungen Menschen nicht mehr wegzudenken. Im Jahr 2022 verbrachten Jugendliche täglich durchschnittlich 204 min ihrer Freizeit mit dem Internet [1], ein Jahr später sogar 224 min [2]. Diese Entwicklung birgt sowohl Chancen als auch Herausforderungen für die öffentliche Gesundheit. Einerseits ermöglicht der schnelle Zugang zu relevanten Informationen, dass Menschen besser informierte Entscheidungen in Bezug auf ihre Gesundheit treffen können. So schätzen Kinder und Jugendliche diese schnelle Verfügbarkeit von Informationen im Internet, um sich über Möglichkeiten zur Verbesserung ihrer Gesundheit zu informieren oder um sich online mit Gleichaltrigen zu vernetzen und in sozialen Medien, auf YouTube oder in Online-Foren emotionale Unterstützung zu finden [3]. Dabei sind für Jugendliche alltägliche Gesundheitsthemen (z. B. Sportverletzungen, chronische Erkrankungen, sexuelle Gesundheit, Fitness, Infektionen) von besonderem Interesse [4]. Gleichzeitig kann die große Menge verfügbarer Informationen überwältigend sein und jungen Menschen Schwierigkeiten bereiten, sich in dieser Informationsflut zurechtzufinden [3]. Die resultierende Gefahr der sogenannten Infodemie erschwert es erheblich, verlässliche Informationen von irreführenden zu unterscheiden [5]. Diese Problematik wird beispielsweise in einer repräsentativen Befragung der Vodafone Stiftung Deutschland deutlich, die sich mit dem Umgang junger Menschen (zwischen 14 und 24 Jahren) mit Falschinformationen während der COVID-19-Pandemie beschäftigte [6]. So kommen etwa 76 % der Befragten mindestens einmal pro Woche mit Falschnachrichten in Kontakt, 28 % davon mindestens einmal täglich. Die Kontakthäufigkeit mit Falschinformationen ist dabei in den letzten Jahren deutlich gestiegen: Während im Jahr 2018 51 % der Befragten mindestens einmal pro Woche mit Falschinformationen konfrontiert waren, stieg diese Zahl 2019 bereits auf 64 % [6]. Problematisch ist dabei, dass 34 % der Befragten Unsicherheiten beim Erkennen von Falschinformationen haben, bei den 14- bis 19-Jährigen sogar 42 % [6].

Um diese Fülle von verfügbaren digitalen Informationen korrekt einordnen und fundierte gesundheitsbezogene Entscheidungen treffen zu können, muss die digitale Gesundheitskompetenz (dGK) von Kindern und Jugendlichen stärker berücksichtigt werden. Die dGK umfasst die Fähigkeit, digitale Informations- und Kommunikationstechnologien effektiv zu nutzen, um gesundheitsbezogene Informationen zu suchen, zu finden, zu verstehen, zu bewerten und anzuwenden, um gesundheitliche Herausforderungen zu bewältigen [7].

Dazu werden insbesondere 7 Dimensionen betrachtet:

- operative Fähigkeiten zur Nutzung digitaler Anwendungen,
- Erstellung eigener Inhalte in digitalen Lebenswelten,
- Fähigkeit, im Internet zu navigieren,
- die richtige Strategie bei der Informationssuche,
- Schutz der Privatsphäre bei der Internetnutzung,
- Bestimmung der Relevanz gefundener Informationen sowie
- Bewertung der Zuverlässigkeit von Informationen aus dem Internet [8, 9].

Dieses Verständnis der dGK fokussiert neben der Bereitstellung von Informationen insbesondere die Interaktion mit digitalen Anwendungen [10]. Erste repräsentative Ergebnisse aus Deutschland ergaben, dass 30,8 % der 11- bis 18-Jährigen ein problematisches und 4,6 % ein unzureichendes Maß der dGK aufwiesen [11]. Dabei zeigten sich die größten Schwierigkeiten in den Items bezogen auf den Schutz der Privatsphäre (47,8–59,4 %), während bei operativen Fähigkeiten kaum Schwierigkeiten bestanden (4,9–6,4 %; [11]). Damit übereinstimmend berichtet eine frühere Studie unter Schülerinnen und Schülern (SuS) der 8. und 9. Klassen von Schwierigkeiten im Umgang mit digitalen Gesundheitsinformationen, insbesondere beim Schutz der Privatsphäre [12]. Beide Studien beschreiben zudem ein niedrigeres Niveau der dGK bei niedrigerem Sozialstatus [11, 12]. Dies deutet darauf hin, dass die dGK ähnlich wie die allgemeine Gesundheitskompetenz [13–17] einem sozialen Gradienten folgt. Angesichts der Bedeutung der dGK für das Gesundheitsverhalten von Kindern und Jugendlichen sind diese Ergebnisse besonders besorgniserregend. Beispielsweise weisen in Deutschland Jugendliche mit mittlerer und geringer dGK ein erhöhtes Risiko für geringe körperliche Aktivität, unregelmäßigen Obstkonsum und täglichen Konsum von zuckerhaltigen Getränken auf [7]. In Israel zeigte sich bei Jugendlichen ein signifikanter Zusammenhang der medienbezogenen Gesundheitskompetenz mit gesunder Ernährung, körperlicher Aktivität, sexueller Aktivität, Sicherheitsverhalten und Substanzkonsum [18]. In China berichteten Jugendliche über eine höhere Intention zu gesundheitsförderlichem Verhalten, wenn sie sich kompetenter in der Informationsbeschaffung sowohl online als auch offline fühlten [19].

Neben der Familie als primärer Sozialisationsinstanz stellt das Setting Schule eine der bedeutsamen Lebenswelten zur Verortung von Inhalten der dGK bei Kindern und Jugendlichen dar [12]. Insbesondere die Vorgabe zur Umsetzung der Strategie „Bildung in der digitalen Welt“ der Kultusministerkonferenz [20], die auf Ebene der Bundesländer in die Medienkompetenzrahmen übersetzt wurde, ermöglicht die Behandlung der dGK als Querschnittsthema über alle Fächer. Ein Beispiel für die Verknüpfung von Maßnahmen zur Förderung der dGK mit dem Medienkompetenzrahmen ist das Forschungsprojekt Tool-HLCA [21, 22]. Dieses Projekt nutzt die zweite Kerndimension „Informieren und Recherchieren“ des Medienkompetenzrahmens Nordrhein-Westfalen, um Unterrichtsmaterialien in Form einer „Toolbox“ zu entwickeln. Diese zielt darauf ab, die Gesundheitskompetenz von SuS effizient und ressourcenschonend zu stärken [21, 22]. Dieser Ansatz zur Förderung der (d)GK in der Schule über die Integration in bestehende Strukturen geht zudem mit den Empfehlungen der Weltgesundheitsorganisation einher [23]. Außerdem bietet die Schule ein ideales Umfeld, um die (d)GK zu fördern, da sie fast alle Kinder im schulpflichtigen Alter erreicht [23, 24]. Somit haben Schulen das Potenzial, individuelle Lernunterschiede auszugleichen und durch soziale Nachteile bedingte Lernunterschiede zu verringern [25]. International zeigte eine kanadische Studie bereits, dass ein spezifisches Interventionsprogramm positiv zur Förderung der dGK in der Schule beitragen kann [26].

Trotz der offensichtlichen Bedeutung des Themas gibt es für Deutschland bisher nur wenige Untersuchungen zum Erlernen der dGK in der Schule. Erste Programme und Lehrmaterialien zur Verknüpfung der Gesundheitskompetenz mit den Medienkompetenzrahmen sind zwar vorhanden [21, 22, 27], dennoch fehlen Ergebnisse, die zeigen, inwieweit Inhalte der dGK in der Schule in Deutschland aus Sicht von SuS tatsächlich erlernt werden. Unabhängig des Gesundheitsbezugs zeigt sich, dass sich 56 % der Jugendlichen mehr schulische Unterstützung zur Erkennung von Falschnachrichten wünschen und die Aufnahme von entsprechenden Inhalten in die Lehrpläne befürworten [6]. Gleichzeitig gaben nur 30 % der 14- bis 24-Jährigen an, dieses Thema an ihrer Schule behandelt zu haben [6].

Angesichts der begrenzten Forschungslage ist das Ziel dieses Beitrags, erste Ergebnisse einer repräsentativen Studie zum Erlernen der dGK in der Schule vorzustellen. Das Hauptaugenmerk liegt auf der Untersuchung, ob SuS in Deutschland Inhalte der dGK in der Schule erlernen. Des Weiteren soll untersucht werden, inwiefern soziodemografische und sozioökonomische Unterschiede hinsichtlich Alter, Geschlecht, Migrationshintergrund und subjektiven sozioökonomischen Status mit dem Erlernen dieser Inhalte zusammenhängen.

## Methoden

### Studiendesign und Stichprobe

Durchgeführt wurde diese Querschnittsstudie in Deutschland unter SuS im Alter von 9 bis 18 Jahren als Teil des von der BARMER Krankenkasse geförderten und umgesetzten Präventionsprojekts „DURCHBLICKT!“. Mithilfe von Lehrmaterialien und modular aufgebauten, *on demand* Lehrerfortbildungen unterstützt das Projekt Lehrkräfte bei der Vermittlung von Inhalten der dGK im Schulunterricht (digital und analog), um SuS zu befähigen, fundierte Entscheidungen im digitalen Gesundheitskontext zu treffen [28]. Die Untersuchung ist Teil der ersten Datenerhebung, die von Oktober bis November 2022 erfolgte. Einschlusskriterium für SuS war ein Wohnort in Deutschland sowie der Besuch einer weiterführenden Schule (Klassen 5 bis 10) zum Zeitpunkt der Erhebung. Die Repräsentativität der Stichprobe wurde auf Schulart und Klassenstufe, Geschlecht, Migrationshintergrund und Verteilung nach Bundesländern hergestellt. Für die Rekrutierung der Teilnehmenden wurden 375 geschulte Jugendinterviewer eines internationalen Marktforschungsinstituts eingesetzt, die jeweils maximal 5 SuS aus ihrem Bekanntenkreis rekrutierten. Die Befragung der Teilnehmenden wurde durch die Jugendinterviewer mithilfe eines standardisierten Fragebogens durchgeführt.

### Instrumente

#### Soziodemografische und sozioökonomische Faktoren

Geschlecht, Alter, Migrationshintergrund und subjektiver Sozialstatus wurden in dieser Studie verwendet. SuS gaben ihr biologisches Geschlecht und Alter an. Für die Datenanalyse wurde das Alter in die Kategorien 9 bis 11 Jahre, 12 bis 15 Jahre und 16 bis 18 Jahre eingeteilt. Der Migrationshintergrund wurde mit 3 Items erfasst, die das Geburtsland der Kinder und Jugendlichen sowie deren Eltern erfassten. Auf Grundlage dieser Informationen wurde zwischen SuS mit Migrationshintergrund (mindestens ein Elternteil oder die SuS selbst außerhalb Deutschlands geboren) und solchen ohne Migrationshintergrund (beide Elternteile und SuS in Deutschland geboren) unterschieden [29]. Die SuS gaben ihren subjektiven Sozialstatus auf einer 5‑Punkte-Likert-Skala an [30], sodass anschließend 3 Kategorien zusammengefasst wurden: niedrig (überhaupt nicht wohlhabend, eher nicht wohlhabend), mittel (durchschnittlich) und hoch (eher wohlhabend, sehr wohlhabend).

#### Erlernen von Inhalten der dGK

Zur Erfassung, ob SuS Inhalte der dGK in der Schule gelernt haben, wurde eine vom Projektteam entwickelte Skala eingesetzt. Die Entwicklung erfolgte entlang der 7 beschriebenen Dimensionen der dGK im Digital Health Literacy Instrument (DHLI; [8]). Dabei wurden die Items einer jeden Dimension des DHLI zusammengefasst und der Schulkontext je Dimension integriert. Im Ergebnis steht eine Skala zur schulischen dGK mit 7 Items, die SuS fragt, inwiefern sie die jeweiligen Fähigkeiten in der Schule erlernen. Dabei werden die Items anhand einer 4‑Punkte-Likert-Skala („gelernt“, „eher gelernt“, „eher nicht gelernt“, „nicht gelernt“) bewertet. Die Skala wurde in einem Pretest mit 12 SuS erprobt und basierend auf den Ergebnissen für die Hauptstudie sprachlich angepasst. Die finalen Items sind in Tab. 1 dargestellt.

**Tab. 1:** Items zur Erfassung des Erlernens der digitalen Gesundheitskompetenz in der Schule

	Item
Operative Fähigkeiten	Ich habe gelernt, den Computer so zu bedienen und Webseiten so zu nutzen, dass ich im Internet nach Informationen rund um das Thema Gesundheit suchen kann (z. B. Computer-Tastatur, Maus oder Links auf Webseiten benutzen)
Erstellung eigener Inhalte	Ich habe gelernt, eine Nachricht rund um das Thema Gesundheit so zu schreiben (z. B. E‑Mail, Chat, Messenger, Blog, Forum), dass andere Personen genau verstehen, was ich meine
Fähigkeit zu navigieren	Ich habe gelernt, mich im Internet gut zurecht zu finden, sodass ich Informationen rund um das Thema Gesundheit suchen und finden kann (z. B. Suchergebnisse filtern, vertrauenswürdige Webseiten finden, zwischen verschiedenen Seiten zu wechseln)
Schutz der Privatsphäre	Ich habe gelernt, wie ich meine persönlichen Gesundheitsdaten und die Daten anderer Menschen im Internet schützen kann (z. B. keine privaten Informationen mit Absicht oder aus Versehen weitergeben oder teilen)
Informationssuche	Ich habe gelernt, wie ich Antworten auf meine Fragen rund um das Thema Gesundheit im Internet finden kann (z. B. gute Suchbegriffe ausdenken, die besten Informationen aussuchen)
Bestimmung der Relevanz	Ich habe gelernt, wie ich die Informationen rund um das Thema Gesundheit aus dem Internet auch in meinem Alltag nutzen und anwenden kann
Bewertung der Zuverlässigkeit	Ich habe gelernt, wie ich die Qualität von Informationen rund um das Thema Gesundheit aus dem Internet kritisch bewerten kann (z. B. entscheiden kann, ob mir nur etwas verkauft werden soll)

### Statistische Analysen

Alle Analysen wurden mithilfe der Statistiksoftware IBM SPSS Statistics 29 (IBM Corp., Armonk, NY, USA) vorgenommen. Zunächst wurden die relativen und absoluten Häufigkeiten über die Ausprägungen hinsichtlich des Erlernens der 7 Dimensionen der dGK in der Schule berechnet. Anschließend wurden soziodemografische und sozioökonomische Unterschiede im Erlernen von Inhalten der dGK untersucht. Dazu wurden Kreuztabellen mit angeschlossenem *χ*^2^-Test (*p* < 0,05) herangezogen. Zur Bestimmung der Effektgröße wurde Cramers V berechnet und nach J. Cohen [31] interpretiert: kleiner Effekt: V = 0,1; mittlerer Effekt: V = 0,3; großer Effekt: V = 0,5. Anschließend wurden 7 binärlogistische Regressionen berechnet, um zu ermitteln, inwieweit Geschlecht, Alter, Migrationshintergrund und subjektiver Sozialstatus mit dem Erlernen der dGK zusammenhängen. In allen Regressionsmodellen lagen die Korrelationen zwischen den Prädiktoren bei r < 0,80, was darauf hinweist, dass Multikollinearität die Analyse nicht beeinflusst hat. Basierend auf den Ergebnissen des *χ* ^2^-Tests wurden folgende Kategorien der Prädiktoren als Referenzkategorien festgelegt: weiblich, 16–18 Jahre, kein Migrationshintergrund, hoher subjektiver Sozialstatus. Zur Prüfung der Signifikanz der Modelle wurden Omnibus-Tests der Modellkoeffizienten (*p* < 0,05) durchgeführt. Zur Bewertung der durch das Modell erklärten Varianz wurde Nagelkerkes R^2^ herangezogen, dessen Varianzaufklärung nach K. Backhaus et al. [32] wie folgt interpretiert wird: *R*^2^ > 0,20 kleiner Effekt, *R*^2^ > 0,40 mittlerer Effekt, *R*^2^ > 0,50 hoher Effekt. Mit dem Hosmer-Lemeshow-Test wurde die Anpassungsgüte des Modells festgestellt (*p* > 0,05), welche bei allen Modellen eine angemessene Modellqualität bestätigte: operative Fähigkeiten *χ*^2^ (8) = 14,67, *p* > 0,05; Erstellung eigener Inhalte *χ*^2^ (8) = 5,41, *p* > 0,05; Fähigkeit zu navigieren *χ*^2^ (8) = 6,24, *p* > 0,05; Schutz der Privatsphäre *χ*^2^ (8) = 9,73, *p* > 0,05; Informationssuche *χ*^2^ (8) = 3,72, *p* > 0,05; Bestimmung der Relevanz *χ*^2^ (7) = 7,56, *p* > 0,05; Bewertung der Zuverlässigkeit *χ*^2^ (8) = 5,05, *p* > 0,05. Der Gesamtprozentsatz korrekter Klassifikation lag bei allen Modellen über dem Cut-off-Wert von 0,50. Ausgewiesen werden die Odds Ratios (OR) als Chancenverhältnisse sowie die dazugehörigen Konfidenzintervalle (95 %-KI).

## Ergebnisse

### Stichprobenbeschreibung

An der Untersuchung nahmen 1448 in Deutschland lebende SuS der Sekundarstufe I teil. Die Geschlechterverteilung war nahezu ausgewogen mit 50,8 % männlichen und 49,2 % weiblichen Teilnehmenden. Das Durchschnittsalter der Stichprobe betrug 12,98 Jahre, wobei die meisten SuS zwischen 12 und 15 Jahre alt waren. Einzelheiten zur Studienpopulation sind Tab. 2 zu entnehmen.

**Tab. 2:** Beschreibung der Stichprobe (*n* = 1448)

	*n*	%
*Gesamt*	1448	100
*Biologisches Geschlecht*		
Männlich	736	50,8
Weiblich	712	49,2
*Alter*		
9–11 Jahre	389	26,9
12–15 Jahre	909	62,8
16–18 Jahre	150	10,4
*Migrationshintergrund*		
Mit Migrationshintergrund	484	33,4
Ohne Migrationshintergrund	964	66,6
*Subjektiver Sozialstatus*		
Niedrig	180	12,4
Mittel	824	56,9
Hoch	444	30,7

### Erlernen dGK in der Schule

Über alle 7 Dimensionen der dGK hinweg gaben etwa 50 % der SuS an, diese Fähigkeiten in der Schule (eher) nicht erlernt zu haben (Tab. 3).

**Tab. 3:** Erlernen von Inhalten der digitalen Gesundheitskompetenz in der Schule

	*n*	In der Schule (eher) erlernt	In der Schule (eher) nicht erlernt
		*n* (%)	*n* (%)
Operative Fähigkeiten	1393	705 (50,6)	688 (49,4)
Erstellung eigener Inhalte	1384	622 (44,9)	762 (55,1)
Fähigkeit zu navigieren	1395	701 (50,3)	694 (49,7)
Schutz der Privatsphäre	1373	692 (50,4)	681 (49,6)
Informationssuche	1392	685 (49,2)	707 (50,8)
Bestimmung der Relevanz	1385	659 (47,6)	726 (52,4)
Bewertung der Zuverlässigkeit	1384	700 (50,6)	684 (49,4)

### Soziodemografische und sozioökonomische Unterschiede beim Erlernen von Inhalten der dGK

In Bezug auf das Erlernen der verschiedenen Dimensionen der dGK in der Schule zeigten sich signifikante Unterschiede in Abhängigkeit von Geschlecht, Alter, Migrationshintergrund und subjektivem Sozialstatus (Tab. 4). Geschlechterunterschiede konnten lediglich beim Erlernen der geeigneten Informationssuche und der Bestimmung der Relevanz von gesundheitsbezogenen Informationen gefunden werden. Hier gaben Schülerinnen häufiger als Schüler an, entsprechende Inhalte in der Schule zu erlernen. Ältere SuS (16 bis 18 Jahre) berichteten häufiger als jüngere SuS (12 bis 15 Jahre; 9 bis 11 Jahre) über das Erlernen von Inhalten zu operativen Fähigkeiten, der Fähigkeiten zu navigieren, zum Schutz der Privatsphäre, zur Informationssuche, zur Bestimmung der Relevanz und zur Bewertung der Zuverlässigkeit. Im Vergleich zu SuS mit Migrationshintergrund berichteten SuS ohne Migrationshintergrund häufiger über das Erlernen operativer Fähigkeiten, der Fähigkeit zu navigieren, der Informationssuche, der Bestimmung der Relevanz und der Bewertung der Zuverlässigkeit. Unterschiede in Bezug auf den subjektiven Sozialstatus wurden bei allen Dimensionen der dGK gefunden, mit Ausnahme der operativen Fähigkeiten. Dabei gaben SuS mit hohem subjektiven Sozialstatus häufiger das Erlernen der entsprechenden Inhalte an als SuS mit mittlerem oder niedrigem Status.

**Tab. 4:** Erlernen von Inhalten der digitalen Gesundheitskompetenz in der Schule nach soziodemografischen Merkmalen

	Operative Fähigkeiten		Erstellung eigener Inhalte		Fähigkeit zu navigieren		Schutz der Privatsphäre		Informationssuche		Bestimmung der Relevanz		Bewertung der Zuverlässigkeit	
	Ja	Nein	Ja	Nein	Ja	Nein	Ja	Nein	Ja	Nein	Ja	Nein	Ja	Nein
	% (*n*)	% (*n*)	% (*n*)	% (*n*)	% (*n*)	% (*n*)	% (*n*)	% (*n*)	% (*n*)	% (*n*)	% (*n*)	% (*n*)	% (*n*)	% (*n*)
*Biologisches Geschlecht*	*n*. s.		*n*. s.		*n*. s.		*n*. s.		X^2^ (1) = 6,86		X^2^ (1) = 6,92		*n*. s.	
									*p* = 0,009, V = 0,01		*p* = 0,009, V = 0,01			
Männlich	49,6 %	50,4 %	43,5 %	56,5 %	48,0 %	52,0 %	48,0 %	52,0 %	45,8 %	54,2 %	44,1 %	55,9 %	49,4 %	50,6 %
	(350)	(356)	(306)	(397)	(340)	(368)	(333)	(361)	(323)	(383)	(311)	(394)	(347)	(356)
Weiblich	51,7 %	48,3 %	46,4 %	53,6 %	52,5 %	47,5 %	52,9 %	47,1 %	52,8 %	47,2 %	51,2 %	48,8 %	51,8 %	48,2 %
	(355)	(332)	(316)	(365)	(361)	(326)	(359)	(320)	(362)	(324)	(348)	(332)	(353)	(328)
*Alter*	X^2^ (2) = 24,29		n. s.		X^2^ (2) = 10,92		X^2^ (2) = 10,34		X^2^ (2) = 9,87 (2)		X^2^ (2) = 15,56		X^2^ (2) = 19,16	
	*p* < 0,001, V = 0,13				*p* = 0,004, V = 0,09		*p* = 0,006, V = 0,09		*p* = 0,007, V = 0,08		*p* < 0,001, V = 0,10		*p* < 0,001, V = 0,12	
9–11 Jahre	41,3 %	58,7 %	39,9 %	60,1 %	43,9 %	56,1 %	43,5 %	56,5 %	42,1 %	57,9 %	40,0 %	60,0 %	41,6 %	58,4 %
	(150)	(213)	(142)	(214)	(159)	(203)	(154)	(200)	(150)	(206)	(144)	(216)	(150)	(211)
12–15 Jahre	52,2 %	47,8 %	46,2 %	53,8 %	51,4 %	48,6 %	52,1 %	47,9 %	51,3 %	48,7 %	48,9 %	51,1 %	52,6 %	47,4 %
	(460)	(422)	(407)	(474)	(455)	(431)	(453)	(417)	(455)	(432)	(430)	(449)	(462)	(416)
16–18 Jahre	64,2 %	35,8 %	49,7 %	50,3 %	59,2 %	40,8 %	57,0 %	43,0 %	53,7 %	46,3 %	58,2 %	41,8 %	60,7 %	39,3 %
	(95)	(53)	(73)	(74)	(87)	(60)	(85)	(64)	(80)	(69)	(85)	(61)	(88)	(57)
*Migrationshintergrund (MH)*	X^2^ (1) = 5,06		*n*. s.		X^2^ (1) = 6,27		*n*. s.		*X*^2^ (1) = 8,14		*X*^2^ (1) = 4,87		*X*^2^ (1) = 6,92	
	*p* = 0,02, V = 0,06				*p* = 0,01, V = 0,7				*p* = 0,004, V = 0,08		*p* = 0,03, V = 0,06		*p* = 0,009, V = 0,07	
Mit MH	46,4 %	53,6 %	42,1 %	57,9 %	45,6 %	54,4 %	47,5 %	52,5 %	43,9 %	56,1 %	43,5 %	56,5 %	45,7 %	54,3 %
	(220)	(254)	(201)	(276)	(218)	(260)	(225)	(249)	(209)	(267)	(207)	(269)	(219)	(260)
Ohne MH	52,8 %	47,2 %	46,4 %	53,6 %	52,7 %	47,3 %	51,9 %	48,1 %	52,0 %	48,0 %	49,7 %	50,3 %	53,1 %	46,9 %
	(485)	(434)	(421)	(486)	(483)	(434)	(467)	(432)	(476)	(440)	(452)	(457)	(481)	(424)
*Subjektiver Sozialstatus*	*n*. s.		*X*^2^ (2) = 8,93		*X*^2^ (2) = 8,47		*X*^2^ (2) = 20,25		*X*^2^ (2) = 13,96		*X*^2^ (2) = 8,02		*X*^2^ (2) = 7,51	
			*p* = 0,01, V = 0,08		*p* = 0,01, V = 0,08		*p* < 0,001, V = 0,12		*p* < 0,001, V = 0,10		*p* = 0,02, V = 0,08		*p* = 0,02, V = 0,07	
Niedrig	46,7 %	53,3 %	40,5 %	59,5 %	45,2 %	54,8 %	44,4 %	55,6 %	44,0 %	56,0 %	41,8 %	58,2 %	50,3 %	49,7 %
	(78)	(89)	(66)	(97)	(75)	(91)	(72)	(90)	(73)	(93)	(69)	(96)	(85)	(84)
Mittel	49,6 %	50,4 %	42,7 %	57,3 % %	48,2 %	51,8 %	46,8 %	53,2 %	46,3 %	53,7 %	45,9 %	54,1 %	47,7 %	52,3 %
	(393)	(399)	(338)	(454)	(383)	(411)	(367)	(418)	(367)	(426)	(360)	(425)	(374)	(410)
Hoch	53,9 %	46,1 %	50,8 %	49,2 %	55,9 %	44,1 %	59,4 %	40,6 %	56,6 %	43,4 %	54,1 %	47,1 %	55,9 %	44,1 %
	(234)	(200)	(218)	(211)	(243)	(192)	(253)	(173)	(245)	(188)	(425)	(205)	(241)	(190)

*Ja* in der Schule (eher) gelernt, *Nein* in der Schule (eher) nicht gelernt, *n.* *s.* nicht signifikant (*p* > 0,05), *V* Cramers V

### Zusammenhänge soziodemografischer und sozioökonomischer Faktoren mit dem Erlernen von Inhalten der dGK

7 binärlogistische Regressionen wurden berechnet, um zu überprüfen, inwieweit die Faktoren Geschlecht (männlich, weiblich), Alter (9–11 Jahre, 12–15 Jahre, 16–18 Jahre), Migrationshintergrund (mit, ohne) und subjektiver Sozialstatus (niedrig, mittel, hoch) dazu beitragen, dass SuS die Inhalte der dGK in der Schule gelernt haben. Alle Regressionsmodelle waren statistisch signifikant (Tab. 5): operative Fähigkeiten *χ*^2^ (6) = 31,48, *p* < 0,001, *R*^2^ = 0,03, *n* = 1393; Erstellung eigener Inhalte *χ*^2^ (6) = 16,84, *p* < 0,05, *R*^2^ = 0,02, *n* = 1384; Fähigkeit zu navigieren *χ*^2^ (6) = 26,60, *p* < 0,001, *R*^2^ = 0,03, *n* = 1395; Schutz der Privatsphäre *χ*^2^ (6) = 34,61, *p* < 0,001, *R*^2^ = 0,03, *n* = 1373; Informationssuche *χ*^2^ (6) = 37,01, *p* < 0,001, *R*^2^ = 0,04, *n* = 1392; Bestimmung der Relevanz *χ*^2^ (6) = 33,25, *p* < 0,001, *R*^2^ = 0,03, *n* = 1385; Bewertung der Zuverlässigkeit *χ*^2^ (6) = 31,72, *p* < 0,001, *R*^2^ = 0,03, *n* = 1384.

**Tab. 5:** Ergebnisse der Regression

	Operative Fähigkeiten	Erstellung eigener Inhalte	Fähigkeit zu navigieren	Schutz der Privatsphäre	Informationssuche	Bestimmung der Relevanz	Bewertung der Zuverlässigkeit
	OR [95 %-KI]	OR [95 %-KI]	OR [95 %-KI]	OR [95 %-KI]	OR [95 %-KI]	OR [95 %-KI]	OR [95 %-KI]
*Biologisches Geschlecht*							
Weiblich (Referenz)	–	–	–	–	–	–	–
Männlich	0,92	0,89	0,84	0,82	0,76	0,75	0,90
	[0,72; 1,14]	[0,72; 1,11]	[0,68; 1,03]	[0,66; 1,02]	[0,61; 0,94]^*^	[0,61; 0,93]^*^	[0,73; 1,12]
*Alter*							
16–18 Jahre (Referenz)	–	–	–	–	–	–	–
12–15 Jahre	0,62	0,90	0,76	0,86	0,95	0,71	0,74
	[0,43; 0,89]^*^	[0,64; 1,29]	[0,53; 1,08]	[0,61; 1,23]	[0,67; 1,36]	[0,50; 1,01]	[0,52; 1,07]
9–11 Jahre	0,41	0,71	0,57	0,62	0,67	0,50	0,49
	[0,27; 0,60]^*^	[0,48; 1,04]	[0,38; 0,84]^*^	[0,42; 0,91]^*^	[0,45; 0,99]^*^	[0,34; 0,74]^*^	[0,33; 0,73]^*^
*Migrationshintergrund (MH)*							
Ohne MH (Referenz)	–	–	–	–	–	–	–
Mit MH	0,80	0,86	0,77	0,860	0,74	0,80	0,77
	[0,64; 1,00]	[0,69; 1,08]	[0,62; 0,96]^*^	[0,69; 1,08]	[0,59; 0,93]^*^	[0,64; 1,00]	[0,61; 0,96]^*^
*Subjektiver Sozialstatus*							
Hoch (Referenz)	–	–	–	–	–	–	–
Mittel	0,88	0,73	0,76	0,61	0,67	0,78	0,74
	[0,69; 1,11]	[0,58; 0,93]^*^	[0,60; 0,96]^*^	[0,48; 0,78]^*^	[0,53; 0,85]^*^	[0,62; 0,99]^*^	[0,59; 0,94]^*^
Niedrig	0,76	0,66	0,66	0,55	0,60	0,65	0,80
	[0,53; 1,09]	[0,46; 0,95]^*^	[0,46; 0,96]^*^	[0,38; 0,79]^*^	[0,42; 0,86]^*^	[0,45; 0,94]^*^	[0,56; 1,15]

*OR* Odds Ratio* KI* Konfidenzintervall

^*^p *<* *0,05*

Die Wahrscheinlichkeit, Inhalte der dGK in der Schule erlernt zu haben, sinkt daher signifikant unter verschiedenen Bedingungen:

- operative Fähigkeiten: bei 9‑ bis 11- und 12- bis 15-Jährigen,
- Erstellen eigener Inhalte: bei niedrigem oder mittlerem subjektiven Sozialstatus,
- Fähigkeit zu navigieren: bei 9‑ bis 11-Jährigen, bei SuS mit Migrationshintergrund sowie bei niedrigem oder mittlerem subjektiven Sozialstatus,
- Schutz der Privatsphäre: bei 9‑ bis 11-Jährigen sowie bei niedrigem oder mittlerem subjektiven Sozialstatus,
- Informationssuche: bei männlichen Schülern, 9‑ bis 11-Jährigen, bei SuS mit Migrationshintergrund sowie bei niedrigem oder mittlerem subjektiven Sozialstatus,
- Bestimmung der Relevanz: bei männlichen Schülern, 9‑ bis 11-Jährigen sowie bei niedrigem oder mittlerem subjektiven Sozialstatus,
- Bewertung der Zuverlässigkeit: bei 9‑ bis 11-Jährigen, SuS mit Migrationshintergrund sowie bei mittlerem subjektiven Sozialstatus.

Alle OR und 95 %-KI können Tab. 5 entnommen werden.

## Diskussion

Dies ist die erste Studie, die repräsentative Ergebnisse zum Status des Erlernens von Inhalten der dGK von SuS der Sekundarstufe I in Deutschland liefert. Etwa die Hälfte aller befragten SuS gab an, Inhalte der dGK in der Schule (eher) nicht zu erlernen. Diese Ergebnisse sind besonders besorgniserregend, wenn man bedenkt, dass etwa 53 % der SuS in Deutschland eine geringe dGK aufweisen [33, 34]. Unter Berücksichtigung der größten Schwierigkeiten für SuS ergibt sich ein besonderer Bedarf an Bildungsinhalten zum Schutz der Privatsphäre, zur Navigationsfähigkeit und für die Bewertung der Zuverlässigkeit von gesundheitsbezogenen Informationen [11, 33]. Zwar lassen die Ergebnisse der vorliegenden Untersuchung mit 49,4 % der SuS, die angeben, operative Fähigkeiten nicht im schulischen Kontext zu erlernen, einen hohen Bedarf vermuten, dennoch muss geklärt werden, ob solche grundlegenden digitalen Fertigkeiten im schulischen Rahmen vermittelt werden müssen. Die nach 1997 geborenen Generationen sind mit Medien, digitalen Technologien und sozialen Netzwerken vertraut und erfahren in deren Umgang, da sie seit ihrer Kindheit bzw. Geburt mit diesen Technologien interagieren [35]. So besitzen bereits 94 % der 12- bis 13-Jährigen und 99 % der 18- bis 19-Jährigen ein Smartphone, das 92 % täglich nutzen [1]. Eine frühere Untersuchung bestätigt zudem, dass operative Fähigkeiten kaum Schwierigkeiten bereiten [11, 33].

Einerseits könnte also argumentiert werden, dass sich Schulen stärker auf Aspekte der dGK konzentrieren sollten, die über die operativen Fähigkeiten hinausgehen und nicht automatisch durch den allgemeinen Umgang mit digitalen Medien erworben werden. Erste Daten aus Deutschland zeigen, dass Lehrkräfte sich in der Vermittlung der dGK auf die Erstellung eigener Inhalte und die Bestimmung ihrer Relevanz fokussieren, während operative Fähigkeiten weniger im Vordergrund stehen [33]. Jedoch können ohne diese grundlegenden Fähigkeiten zur Bedienung digitaler Endgeräte andere Inhalte der dGK weder erlernt noch angewendet werden. Die Herausforderung besteht darin, ein Gleichgewicht zwischen der Vermittlung grundlegender digitaler Fähigkeiten und spezialisierter dGK zu finden. Zukünftige Forschung sollte untersuchen, in welchem Umfang und in welchen Bereichen Jugendliche grundlegende digitale Fähigkeiten erwerben und wie das Erlernen von dGK-Inhalten in der Schule das Niveau der dGK beeinflusst.

Um den Bedarf an Maßnahmen zur Vermittlung von Inhalten der dGK gezielt zu ermitteln, wurde der Fokus dieser Untersuchung auf soziodemografische und sozioökonomische Faktoren gelegt. Stratifiziert nach diesen Merkmalen lassen sich Unterschiede im Erlernen der Inhalte der dGK feststellen. Die Chi-Quadrat-Tests identifizierten hierbei erste signifikante Unterschiede. Allerdings bieten die Regressionsmodelle durch die gleichzeitige Betrachtung mehrerer Einflussfaktoren ein detaillierteres und aussagekräftigeres Bild im Zusammenspiel dieser Merkmale.

Zusammenfassend geben Schüler im Vergleich zu Schülerinnen an, weniger Inhalte bezüglich der Informationssuche und der Bestimmung der Relevanz in der Schule zu erlernen. Frühere Untersuchungen fanden heterogene Befunde hinsichtlich des Niveaus der dGK bei Jungen und Mädchen. Eine repräsentative Studie fand kein erhöhtes Risiko für ein niedrigeres Niveau der dGK, weder bei Mädchen noch bei Jungen [11]. Dennoch konnten in Deutschland bereits geschlechterspezifische Unterschiede zuungunsten der Mädchen in den Bereichen Informationssuche, Erstellung eigener Inhalte sowie Bewertung der Zuverlässigkeit festgestellt werden [12]. Insofern könnte die in dieser Untersuchung vorliegende höhere Wahrscheinlichkeit zum Erlernen der Informationssuche und der Bestimmung der Relevanz in der Schule bei Mädchen dazu beitragen, diese Unterschiede im Niveau der dGK zu kompensieren.

SuS mit Migrationshintergrund sind in den Bereichen Bewertung der Zuverlässigkeit, Navigationsfähigkeit und Informationssuche einem höheren Risiko ausgesetzt, diese Inhalte in der Schule nicht zu erlernen. Währenddessen wurde der Migrationshintergrund als Faktor identifiziert, der das Risiko eines niedrigeren Niveaus der Navigationsfähigkeit, der Informationssuche, der Erstellung eigener Inhalte sowie der Bewertung der Zuverlässigkeit erhöht [11].

Zudem ließ sich ein sozialer Gradient im Erlernen der dGK über alle Dimensionen erkennen, mit Ausnahme der operativen Fähigkeiten. Somit gaben SuS mit niedrigerem subjektiven Sozialstatus mit geringerer Wahrscheinlichkeit an, Inhalte der dGK in der Schule zu erlernen. Vor dem Hintergrund, dass auch das Niveau der dGK einem sozialen Gradienten folgt [11, 12, 34], ist die Vermittlung der dGK in der Schule auch zur Förderung der sozialen Chancengleichheit von Relevanz. Dies wäre auch im Hinblick auf den sozialen Gradienten der allgemeinen Gesundheitskompetenz vorteilhaft [13, 14, 17]. Weitere Relevanz zeigt sich unter Beachtung, dass während der COVID-19-Pandemie besonders junge Menschen mit niedrigerem Bildungshintergrund Schwierigkeiten hatten, glaubwürdige Nachrichten von unglaubwürdigen zu unterscheiden, und es eben diejenigen waren, die seltener das Thema Falschnachrichten im Unterricht behandelten [6].

Zuletzt zeigt sich, dass jüngere SuS einem größeren Risiko ausgesetzt sind, die Inhalte der dGK nicht in der Schule zu erlernen, mit Ausnahme der Fähigkeit zur Erstellung eigener Inhalte. Bezugnehmend auf das Niveau der dGK, das bei jüngeren SuS niedriger ausfällt [11, 33, 34], unterstreichen die Ergebnisse die Notwendigkeit, frühzeitig und altersgerecht Themen der dGK in den Lehrplan zu integrieren, um jüngere SuS besser zu erreichen. Die JIM-Plus-Studie 2022 zeigt ebenfalls, dass ältere Jugendliche häufiger Falschnachrichten überprüfen als jüngere [36]. Gleichzeitig berichten mehr ältere SuS, dass ihre Lehrkraft das Thema Falschnachrichten in den Unterricht integrierte [36]. Andererseits können die Ergebnisse, dass Heranwachsende mit steigendem Alter vermehrt vom Erlernen der dGK in der Schule berichten, ermutigen. Sie deuten darauf hin, dass deren Inhalte im Laufe der Schulzeit progressiv vermittelt und integriert werden. Weitere Forschung muss klären, wie genau diese Inhalte über die Schuljahre hinweg aufgebaut und verstärkt werden und welche pädagogischen Ansätze dabei erfolgreich sind.

### Stärken und Limitationen

Die vorliegende Studie liefert erste repräsentative Ergebnisse zum Erlernen von Inhalten der dGK in der Schule bei SuS der Sekundarstufe I und leistet somit einen Beitrag zur Schließung der bestehenden Forschungslücke. Mit insgesamt 1448 Teilnehmenden ermöglicht die Stichprobe eine umfassende Repräsentation der Zielpopulation, was die Verallgemeinerbarkeit unserer Ergebnisse unterstützt. Die *Peer-to-Peer-*Erhebungsmethode durch geschulte, jugendliche Interviewende unter Zuhilfenahme des strukturierten Fragebogens ermöglichte es den Teilnehmenden, Verständnisfragen während der Befragung zu adressieren. Nichtsdestotrotz basiert die Untersuchung auf selbstberichteten Daten, die aufgrund ihres subjektiven Charakters und der damit verbundenen Risiken der sozialen Erwünschtheit und des Erinnerungsfehlers potenziellen Verzerrungen ausgesetzt sind. Dahin gehend muss auch hinterfragt werden, ob der spezifische Gesundheitsbezug des Erhebungsinstruments die Ergebnisse beeinflusst hat. Beispielsweise könnte dies zu einer progressiveren Bewertung des Umfangs der vermittelten Inhalte geführt haben, obwohl die grundlegenden Aspekte der Medienkompetenzbildung bereits thematisiert wurden. Entsprechend sollte zukünftige Forschung klären, wie die Förderung der Medienkompetenz die dGK beeinflusst und welche konkreten Lehr- und Lernstrategien entwickelt werden können, um beide Bereiche effektiv zu stärken. Dabei könnten zunächst bereits vorhandene Programme, wie beispielsweise aus Tool-HLCA [21, 22], implementiert und dahin gehend evaluiert werden, inwiefern beide Konstrukte zusammenhängen. Des Weiteren muss beachtet werden, dass das Querschnittsdesign der Studie keine kausalen Rückschlüsse erlaubt. Bei der Betrachtung der Regressionsmodelle fallen insbesondere die niedrigen R^2^-Werte auf. Dies bedeutet, dass die Modelle nur einen kleinen Teil der Varianz in den abhängigen Variablen erklären können. Zukünftige Forschung sollte daher weitere Variablen identifizieren und in das Modell integrieren, die potenziell einen stärkeren Einfluss auf das Erlernen der dGK in der Schule haben könnten. Optimalerweise sollten dabei nicht nur Variablen auf individueller Ebene der SuS mit einbezogen werden, sondern auch solche, die das Lehrpersonal und die Schulumgebung betreffen. Das Einbeziehen dieser Perspektiven kann gleichzeitig dazu beitragen, die potenziellen Verzerrungen infolge der selbstberichteten Daten besser einschätzen zu können. Hier muss beachtet werden, dass 42 % der befragten Lehrkräfte einer Studie aus Deutschland ebenfalls Schwierigkeiten in der Beschaffung von und im Umgang mit digitalen Gesundheitsinformationen haben [37]. Somit gilt es zu klären, inwieweit die dGK der Lehrkräfte mit dem Vermitteln und Erlernen der Inhalte der dGK zusammenhängen. Des Weiteren können binäre Regressionsanalysen auch zu Informationsverlusten und einer Unterschätzung des Ausmaßes der Ergebnisvariation zwischen den Gruppen führen, weshalb lineare Regressionsanalysen mit kontinuierlichen Daten für weitere Analysen benötigt werden.

## Fazit

Diese Studie ist die erste, die repräsentative Ergebnisse zum Erlernen dGK bei SuS in Deutschland liefert. Sie zeigt, dass etwa die Hälfte der SuS diese Inhalte nicht in der Schule erlernt, was angesichts ihrer geringen dGK besorgniserregend ist. Geschlechter- und Migrationshintergrundunterschiede beeinflussen das Erlernen der dGK in der Schule ebenso wie das Alter und der subjektive Sozialstatus. Die Ergebnisse verdeutlichen die Notwendigkeit zielgruppenspezifischer Bildungsstrategien, um Inhalte der dGK in bestehende curriculare Konzepte zu integrieren. Die digitale Bildung muss, wie vom Bundesministerium für Bildung und Forschung fokussiert, weiterhin verstärkt in den Schulen verankert werden. Solche Strategien können maßgeblich zur Verringerung sozialer Ungleichheiten beitragen, insbesondere wenn Programme geschlechts- und migrationsspezifisch ausgerichtet sind und gezielt jüngere SuS sowie solche mit niedrigem sozioökonomischen Status adressieren. Dennoch muss geklärt werden, ob ein höherwertiges Lernen in der Schule letztlich auch mit einem höheren Niveau der dGK einhergeht. Daran anknüpfend sollte zukünftige Forschung klären, inwiefern Medien- und Digitalkompetenz mit der dGK zusammenhängen, um effektive und nachhaltige Programme zu entwickeln.
